# Role of ethno-phytomedicine knowledge in healthcare of COVID-19: advances in traditional phytomedicine perspective

**DOI:** 10.1186/s43088-022-00277-1

**Published:** 2022-08-04

**Authors:** Md Nasir Ahmed, Kerry Hughes

**Affiliations:** 1 Independent Researcher, Dhaka, Bangladesh; 2 Ethnopharm LLC, San Luis Obispo, CA USA

**Keywords:** Traditional medicine, Ethnomedicine, Phytomedicine, Medicinal plants, SARS-CoV-2, Ethno-phytomedicine, Immunomodulation, Gut microbiome

## Abstract

**Background:**

Since the outbreak of the COVID-19 virus, ethnomedicinal plants have been used in diverse geographical locations for their purported prophylactic and pharmacological effects. Medicinal plants have been relied on by people around the globe for centuries, as 80% of the world’s population rely on herbal medicines for some aspect of their primary health care needs, according to the World Health Organization.

**Main body:**

This review portrays advances in traditional phytomedicine by bridging the knowledge of ethno-phytomedicine and COVID-19 healthcare. Ethnomedicinal plants have been used for symptoms related to COVID-19 as antiviral, anti-infective, anti-inflammatory, anti-oxidant, antipyretic, and lung–gut immune boosters. Traditionally used medicinal plants have the ability to inhibit virus entry and viral assembly, bind to spike proteins, membrane proteins, and block viral replications and enzymes. The efficacy of traditional medicinal plants in the terms of COVID-19 management can be evaluated by in vitro, in vivo as well as different in silico techniques (molecular docking, molecular dynamics simulations, machine learning, etc.) which have been applied extensively to the quest and design of effective biotherapeutics rapidly. Other advances in traditional phytomedicines against COVID-19 are controlled clinical trials, and notably the roles in the gut microbiome. Targeting the gut microbiome via medicinal plants as prebiotics is also found to be an alternative and potential strategy in the search for a COVID-19 combat strategy.

**Conclusions:**

Since medicinal plants are the sources of modern biotherapeutics development, it is essential to build collaborations among ethnobotanists, scientists, and technologists toward developing the most efficient and the safest adjuvant therapeutics against the pandemic of the twenty-first century, COVID-19.

## Background

COVID-19 is a pandemic disease of the twenty-first century declared by the World Health Organization and caused by a zoonotic virus, known as severe acute respiratory syndrome coronavirus 2 (SARS-CoV-2), which created a catastrophe worldwide with its spread in late 2019. SARS-CoV-2 causing COVID-19 disease produces several symptoms in the human body, which are categorized by the World Health Organization under most common symptoms (fever, dry cough, tiredness), less common symptoms (aches and pains, sore throat, diarrhea, conjunctivitis, headache, loss of taste or smell, rash on skin), and serious symptoms (shortness of breath or difficulty breathing, chest pain or pressure, loss of speech or movement).

It is of great interest that the World Health Organization has been supporting research into traditional medicines for COVID-19 in Africa [121]. Beyond their direct use for COVID-19, phytomedicine or medicinal plants are also used as dietary supplements for supportive therapy to improve immunity against viral infections [50]. In Egypt, about 1/3 of 301 adult participants have mentioned using vitamins/zinc and immune-boosting drinks [63], made from traditionally used medicinal plants to reduce/prevent COVID-19 severity [89]. In China, the USA, and South Asia, the demand for medicinal plants has increased due to the SARS-CoV-2 epidemic [112], and 91.50% of the Chinese COVID-19 cases have used traditional Chinese medicine that may have resulted significantly in improving symptoms, reducing mortality and reinfection rates [70]. Concomitantly, research into phytomedicines and foods and COVID-19 has exploded.

Yang et al. have demonstrated that plant-based foods as alternative medicine can possess immunomodulatory and antiviral properties for the possible preventive strategy against COVID-19 [128]. In England, East Kent Hospitals researchers are trialing Sambucol Black Elderberry liquid as a treatment for COVID-19 [86]. Plant-based functional foods such as *Allium cepa* L., *Allium sativum* L., *Curcuma longa* L., *Camellia sinensis* (L.) Kuntze, *Glycine max* (L.) Merr., *Mangifera indica* L., and *Abelmoschus esculentus* (L.) Moench and plant spices such as *Cinnamomum cassia* (L.) J.Presl, *Piper nigrum* L., and herbs such as *Ocimum basilicum* L., and *Tinospora cordifolia* (Lour.) Merr. have been reported for potential antiviral and immunostimulatory properties against COVID-19 [42]. In a preliminary inventory study, Pieroni et al. have provided a detailed overview of 193 plant species as “foods medicine” that are used in the 17 countries, regions, or cities for COVID-19 healthcare [80].

KTF (Korean Traditional foods) prepared with edible plants, herbs, and mushrooms have the potential to target ACE2 receptors or may disrupt any step of a virus infection pathway, improve gut–lung immunity, and support strong respiratory immunity, and KTF also have protective and immunomodulatory effects in preclinical and clinical studies, reviewed by Das et al. [39]. Bellik et al. [23] have reviewed and demonstrated the roles of micronutrients and phytochemicals based on evidence from in vitro studies and controlled clinical trials against COVID-19 symptoms.

Singirikonda et al. identified edible dry fruits and seeds with demonstrated immune stimulation and antiviral efficacy as potential agents against COVID-19. These were seeds of *Prunus amygdalus* Batsch, fruits of *Phoenix dactylifera* L., fruits of *Juglans nigra* L., nuts of *Arachis hypogea* L., nuts of *Corylus avellana* L., seeds of *Helianthus annuus* L., fruits of *Carya illinoinensis* (Wangenh.) K.Koch, fruits of *Castanea sativa* Mill., fruits of *Pistacia Vera* L., nuts of *Anacardium occidentale* L., seeds of *Prunus armeniaca* L., fruits of *Vitis vinifera* L., fruits of *Prunus domestica* L., fruits of *Ficus carica* L., nuts of *Euryale ferox* Salisb., seeds of *Linum usitatissimum* L., seeds of *Salvia hispanica* L., seeds of *Cucurbita pepo* L., nuts of *Areca catechu* L., and kernel of *Cocos nucifera* L. [99]. In addition, some medicinal herbs such as the powder of *Curcuma longa* L., seeds of *Trachyspermum ammi* (L.) Sprague, peel of *Allium sativum* L., and resin of *Styrax benzoin* and *Boswellia* species can be used as traditional fumigations to disinfect the environment [84].

For this review, we accumulated and summarized ethnomedicinal plant knowledge reported from the diverse geographical locations in the treatment and prevention of coronavirus disease (COVID-19); the literature search has conducted and collected until the end of June 2022 from the WHO COVID-19 database (https://search.bvsalud.org/global-literature-on-novel-coronavirus-2019-ncov/). Other relevant scientific literature available on Google Scholar, Semantic Scholar, and PubMed as well as the WHO COVID-19 database has been evaluated to perform further associated analysis. The use of traditional medicinal plants in the treatment of COVID-19-associated symptoms and clinical, preclinical, in vitro, in vivo*,* and in silico antiviral efficacy of traditional phytomedicines against SARS-CoV-2 has been selected for the review. This review aims to portray a bridge between advances in ethno-phytomedicine and COVID-19 healthcare. Further to be noted, the accepted botanical name was confirmed in the World Flora Online (http://www.worldfloraonline.org/) and in NCBI Taxonomy Browser (https://www.ncbi.nlm.nih.gov/taxonomy).

## Main text

### Ethnomedicine in COVID-19 management from diverse geographical locations

Ethnomedicine is a study of traditional medicine of particular culture that associates ideas and behaviors of how to treat illness and keep healthy [83]. The knowledge of plant-based ethnomedicine or traditional medicine and their derivatives is pivotal for the discovery of new drugs [92] as well as beneficial to treating or preventing coronavirus disease (COVID-9) and related infectious symptoms [56]. The knowledge of ethnomedicine is usually inherited verbally by people living in a culture [92]. Ethnomedicines include medicinal plants used for the prophylactic or treatment of coronavirus disease notably because of their purported antiviral, anti-pyretic, anti-inflammatory, anti-oxidant, and immunostimulatory activities, as well as respiratory immunity-boosting activities. Bioactive compounds in medicinal plants can also enhance membrane integrity to prevent viral entry by binding to envelope proteins, regulating ion channels, and modulating enzymes [75]. The active binding sites of SARS-CoV-2 that can be potential drug targets are chymotrypsin-like protease, RNA-dependent RNA polymerase, hemagglutinin esterase, papain-like protease, 2′-O-methyltransferase, helicase, spike receptor-binding domain, ACE2 receptor, and glucose-regulated proteins [73].

Ethno-phytomedicines or traditional medicinal plants are promising for the alternative management of SARS-CoV-2 as they can block and bind to ACE2 receptors, SARS-CoV helicase, and TMPRSS2 as well as inhibit SARS-CoV-2 entry and replication, reviewed by Ugwah-Oguejiofor and Adebisi [109]. Phytochemicals in medicinal plants have also been demonstrated to exhibit several mechanisms against coronaviruses by targeting virus entry inhibition, viral replication enzymes inhibition, and blockage of virus release activity as well as inhibition of SARS-CoV-2 spike protein, PL^pro^, and 3CL^pro^ [65, 72]. Examples include emodin and baicalin, which can target spike protein, and Iguesterin, Cryptotanshinone, Silvestrol, and Sotetsuflavone, which can target 3CL^pro^, PL^pro^, helicase, and RDRP, respectively [27]. Lectins (mannose-specific) can inhibit binding of SARS-CoV-2 to host cell by targeting the glycans of SARS-COV-2 spike glycoprotein [6]. Table 1 lists some potential SARS-CoV-2 inhibitory phytochemicals.

**Table 1:** A list of phytochemicals reported for potential SARS-CoV-2 inhibitory properties (compiled from [82])

Phytochemical name	Plant source	Potential SARS-CoV-2 inhibitory effects
Emodin	*Rheum palmatum* L.	Inhibiting the SARS-CoV S protein–ACE2 interaction
Luteolin	*Rhodiola kirilowii* (Regel) Maxim.	
Quercetin	*Allium cepa* L.	
Tetra-O-galloyl-b-D-glucose (TGG)	*Galla chinensis*	
3′-(3-Methylbut-2-enyl)-30,4,7-trihydroxyflavane, Broussochalcone and Broussoflavan, Kazinol	*Broussonetia papyrifera* (L.) L'Hér. ex Vent.	Inhibiting the SARS-CoV 3CL^pro^ activity
4-Hydroxyderricin, Isobavachalcone, Xanthoangelol	*Angelica keiskei* (Miq.) Koidz	
Betulinic acid	*Breynia fruticosa* (L.) Müll.Arg.	
Dihydrotanshinone I, Methyl tanshinonate, Rosmariquinone, Tanshinone	*Salvia miltiorrhiza* Bunge	
Hesperetin	*Isatis indigotica* Fortune ex Lindl.	
Hirsutenone	*Alnus japonica* (Thunb.) Steud.	
Isoliquiritigenin	*Glycyrrhiza Glabra* L.	
Quercetin	*Allium cepa* L.	
Quercetin-3-b-galactoside	*Machilus Zuihoensis *Hayata	
Savinin	*Chamaecyparis obtusa* var. *formosana*	
3′0-O-Methyldiplacol, 3′0-O-Methyldiplacone, 4′0-O-Methyldiplacol, 4′0-O-Methyldiplacone, 6-Geranyl-40,5,7-trihydroxy-30,50-Dimethoxyflavanone, Diplacone, Mimulone, Tomentin	*Paulownia Tomentosa* Steud.	Inhibiting the SARS-CoV PL^pro^ activity
4′0-O-Methylbavachalcone, Isobavachalcone, Neobavaisoflavone, Psoralidin	*Psoralea Corylifolia* L.	
Broussochalcone, Biphenyl Propanoids, Papyriflavonol	*Broussonetia papyrifera* (L.) L'Hér. ex Vent.	
Cryptotanshinone, Dihydrotanshinone, Methyl tanshinonate, Tanshinone	*Salvia miltiorrhiza* Bunge	
Curcumin	*Curcuma longa* L.	
Hirsutanonol, Hirsutenone, Rubranol, Rubranoside	*Alnus japonica* (Thunb.) Steud	
Isoliquiritigenin	*Glycyrrhiza Glabra* L.	
Kaempferol	*Zingiber officinale* Roscoe	
Xanthoangelol	*Angelica keiskei* (Miq.) Koidz	
Terrestrimine	*Tribulus terrestris* L.	
Myricetin	*Camellia sinensis* (L.) Kuntze	Inhibiting the SARS-CoV helicase activity
Quercetin	*Allium cepa* L.	
Scutellarein	*Scutellaria Baicalensis* Georgi	

Shoaib and coauthors have summarized various ethnomedicinal plants and their phytochemicals to analyze the potency of COVID-19 treatment and prevention [95]. The plant species *Lycoris radiata* (L'Hér.) Herb., *Artemisia annua* L., *Pyrrosia lingua* (Thunb.) Farw., and *Lindera aggregata* (Sims) Kosterm. showed the median effective concentration = 2.4–88.2 μg/mL against anti-SARS-CoV [95]. Further noted, medicinal plants are the source of antiviral bioactive compounds against SARS-CoV-2 infections that can be determined by in vivo and in vitro assays [24]. Extracts of two medicinal plants that are used in Colombian traditional medicine, *Gliricidia sepium* (Jacq.) Walp., and *Piper tuberculatum* Jacq. showed in vitro anti-SARS-CoV-2 activity [47].

In another study, Hafez Ghoran and coauthors summarized information on ethnomedicinal plants and demonstrated their phytochemical constituents for the potential discovery of novel therapeutics against COVID-19 [53]. Another review study has reported 93 plant-derived antiviral compounds that are potential for the treatment of COVID-19 [3]. Since the outbreak of SARS-CoV-2, many countries from diverse cultures have shared their knowledge of ethnomedicinal plants for the prospective treatment and prevention of COVID-19, and Table 2 compiles ethnomedicinal plants used against COVID-19 and related symptoms reported from diverse geographical locations.

**Table 2:** List of ethnomedicinal plants used for the potential prophylactic and treatment strategy against SARS-CoV-2 causing COVID-19 and associated symptoms, reported from diverse geographical locations (most cited species are marked bold)

Country of traditional medicine	Total number of documented plants	Most commonly used Plants against COVID-19 symptoms	References
Morocco	23	***Allium Sativum* L**.*, Olea europaea* L.*, Allium cepa* L.*, ****Zingiber officinale* Roscoe***, Thymus maroccanus* Ball*, ****Eucalyptus globulus* Labill**.*, Foeniculum vulgare* Mill.*, Curcuma xanthorrhiza* Roxb., *Phoenix dactylifera* L.*, Rosmarinus officinalis* L.*, Thymus satureioides* Coss. & Balansa*, Mentha pulegium* L., and *Pimpinella anisum* L	El Alami et al. [44]
Fez city, Northern Morocco	49	***Syzygium aromaticum (L.)* Merr. & L.M.Perry**, *Thymus vulgaris* L., ***Eucalyptus globulus* Labill**.*, and ****Artemisia vulgaris *L**	Benkhaira et al. [25]
North-Western Morocco	20	***Eucalyptus globulus* Labill**.*, ****Azadirachta indica* A.Juss.***,* and *Ziziphus lotus* (L.) Lam	Chaachouay et al. [32]
Nepal	60	***Zingiber officinale* Roscoe** and *Curcuma angustifolia* Dalzell & A.Gibson	Khadka et al. [64]
Thailand	491	For fever: *Strobilanthes cusia* (Nees) Kuntze*, Acorus calamus* L.*, Melicope glomerate* (W. G. Craib) T.G. Hartley*, ****Andrographis paniculata* (Burm.f.) Nees***, Teucrium viscidum* Blume*, Bidens biternate* (Lour.) Merr. & Sherff*, Paederia pilifera* Hook.f.*, Mimosa pigra* L.*, Monosis parishii* (Hook.f.) H.Rob. & Skvarla***, Artemisia vulgaris* L.***;* For cough: ***Zingiber officinale* Roscoe***, Blumea balsamifera* (L.) DC.*, Elephantopus scaber* L.*, ****Andrographis paniculata* (Burm.f.) Nees***, Teucrium viscidum* Blume*, Pseudelephantopus spicatus* (B. Juss. Ex Aubl.) Rohr ex CFBaker*, Hedyotis acutangula* Champ. ex Benth., *Cyclea barbata* Miers*, Alpinia malaccensis* (Burm.f.) Roscoe*;* For diarrhea: ***Psidium guajava* L.***, Zingiber ottensii* Valeton*, Cassia fistula* L.*, and Aster indicus* var. pinnatifidus*;* *Verbena officinalis* L., and *Dichrocephala integrifolia* (L.f.) Kuntze (L.f.) Kuntze were used to treat skin rash; ***Artemisia vulgaris* L.** was used to treat headache, and *Pothos scandens* L. was used to treat muscle pain	Phumthum et al. [79]
Ethiopia	32 (herbal and non-herbal medicinal products)	Garlic (***Allium sativum* L.**) ginger (***Zingiber officinale* Roscoe)**, lemon (***Citrus limon* (L.) Osbeck**), garden cress (*Lepidium sativum* L.) and Damakase (*Ocimum lamiifolium Hochst*. ex Benth.)	Umeta Chali et al. [110]
Peru	17 (used in the treatment of respiratory symptoms during the COVID-19 pandemic	***Eucalyptus globulus* Labill.***, Piper aduncum* L.*, ****Zingiber officinale* Roscoe***, ****Allium sativum* L.***, Matricaria recutita* L	Villena-Tejada et al. [115]
Cameroon	29	*Citrus aurantiifolia* (Christm.) Swingle*, ****Zingiber officinale* Roscoe***,* and ***Allium sativum* L**	Tsouh Fokou and Youmsi (2020)
Iran	8	*Zataria multiflora* Boiss*, Althaea officinalis* L.*, Hordeum vulgare* L.*, Malva sylvestris* L.*, Matricaria chamomilla var.* coronata J.Gay ex Boiss.*, Glycyrrhiza glabra* L.*, ****Allium sativum* L.,** *and ****Zingiber officinale* Roscoe**	Azimi et al. [19]
African traditional medicine	15	*Abrus precatorius* L.*, Achyranthes aspera* L.*, ****Allium sativum* L.***, Annona muricate* L., *Artemisia afra* Jacq. ex Willd.*, ****Azadirachta indica* A.Juss.***, Cryptolepis sanguinolenta* (Lindl.) Schltr.*, ****Curcuma longa* L.***, ****Euphorbia hirta* L.***, Garcinia kola* Heckel*, ****Glycyrrhiza glabra* L.***, Moringa oleifera* Lam*, ****Nigella sativa* L**.*, ****Psidium guajava* L.***, ****Zingiber officinale* Roscoe**	Adeleye et al. [2]
Algeria (3 provinces, namely Bejaia, Bouira, and Boumerdes)	25	***Syzygium aromaticum* (L.)Merr. & L.M. Perry***, Origanum vulgare* L.*, Mentha* × *piperita* L.*, ****Zingiber officinale* Roscoe***, Aucklandia costus* Falc., ***Artemisia herba-alba* Asso***, Aloysia citriodora* Palau*, ****Eucalyptus globulus*** Labill*, ****Nigella sativa* L.***,* and ***Citrus* × *limon* (L.) Osbeck**	Belmouhoub et al. [22]
Southwest Algeria	2	***Artemisia herba-alba* Asso**, and *Thymus algeriensis* Boiss. & Reut	Bouafia et al. [31]
Bangladesh	15	***Andrographis paniculata* (Burm.f.) Nees***, ****Mangifera indica* L.***, Centella asiatica* (L.) Urb.*, Kalanchoe pinnata* (Lam.) Pers.*, Ocimum sanctum* L.*, Cinnamomum verum* J.Presl*, Laurus nobilis* L.*, Musa* x *sapientum* L.*, ****Syzygium aromaticum* (L.) Merr. & L.M.Perry***, Averrhoa carambola* L.*, ****Nigella sativa* L.***, ****Citrus limon* (L.) Osbeck***, Citrus* x *aurantium* L.*, ****Camellia sinensis* (L.) Kuntze***, ****Zingiber officinale* Roscoe**	Hossain et al. [57]
Belgium (Congolese community)	13	*Artemisia annua* L.*, Citrus spp.,* and ***Zingiber officinale* Roscoe**	De Meyer et al. [40]
North India	24	*Abutilon indicum* (L.) Sweet, ***Allium sativum* L.**, *Aloe vera* (L.) Burm. F, ***Andrographis paniculata* (Burm. f.) Nees**, *Asparagus racemosus* Willd, ***Azadirachta indica* A.Juss.**, *Boerhaavia diffusa* L., ***Camellia sinensis* (L.) Kuntze**, ***Citrus limon* (L.) Osbeck**, ***Curcuma longa* L.**, ***Euphorbia hirta* L.**, *Ficus benghalensis* L., ***Glycyrrhiza glabra* L.**, ***Mangifera indica* L.**, ***Nigella sativa* L.**, *Ocimum tenuiflorum* L., *Phyllanthus emblica* L., *Piper nigrum* L., *Punica granatum* L., *Solanum nigrum* L., *Terminalia chebula* Retz, *Tinospora cordifolia* (Willd.) Miers, *Withania somnifera* (L.) Dunal, ***Zingiber officinale* Roscoe**	Sharma and Rani [93]
UK herbal medicine (typically includes Western herbal medicine, Ayurveda and Traditional Chinese Medicine)	59	***Glycyrrhiza glabra* L.**, *Echinacea spp.*, and ***Andrographis paniculata* (Burm.f.) Nees**	Frost et al. [49]

#### Asian ethnomedicinal plants in COVID-19 healthcare

In Bangladesh, 15 medicinal plants (see Table 2) are reported in case studies to have been used as traditional home remedies for COVID-19 by people who tested positive for SARS-CoV-2 infection. Preparation of these plant species such as *Cinnamomum verum* J.Presl*, Laurus nobilis* L.*,* and *Syzygium aromaticum* (L.) Merr. & L.M.Perry included being boiled in water and the vapor inhaled; certain edible species were used to prepare tea; and consumed as food, especially sour fruits such as *Averrhoa carambola* L.*, Citrus* x *aurantium* L.*, Citrus limon* (L.) Osbeck*,* and *Mangifera indica* L. [57].

As reported in Table 2, in Nepal, a total of 60 plant taxa have been cataloged that were used for the symptoms of COVID-19; whereas, in Thailand was the highest cataloged species with a total of 491 plant species, mainly reported to be used against COVID-19-related symptoms such as fever, cough, diarrhea, skin ash, headache, and muscle pain [64]. Medicinal plants such as *Eurycoma longifolia* Jack, *Coscinium fenestratum* (Gaertn.) Colebr., and *Eleutherine bulbosa* (Mill.) Urb. have been used traditionally for stimulating immunity during the COVID-19 pandemic in Kalimantan, Indonesia [71].

The Adi community residing in Arunachal Pradesh, India, has reported the traditional uses of 39 plant species against asthma, bronchitis, cough, sinusitis, diabetes, malaria, typhoid, and jaundice in the search for immune-stimulatory agents against COVID-19 [97]. Another study has reviewed a total of 55 ethnomedicinal plants of the Himalayas that were used against bronchitis which is a respiratory tract infection caused by influenza virus, rhinovirus, adenovirus, coronavirus, and respiratory syncytial virus; out of 55 only 6 plants (*Hyoscyamus niger* L., *Justicia adhatoda* L., *Ocimum basilicum* L., *Plantago major* L., *Verbascum Thapsus* L., *Zingiber officinale* Roscoe) have reported in vitro antiviral activity against viruses causing bronchitis [11].

#### African ethnomedicinal plants in COVID-19 healthcare

South African traditional medicinal plants have been used for COVID-19 symptoms such as coughs, colds, and fever and have exhibited antiviral activity against coronaviruses or similar viruses [114]. Chikowe and coauthors reviewed and listed 306 Malawi medicinal plants, of which 127 plants have been implicated in management of at least one of the COVID-19-related symptoms or used for against viral infections. Among 127 medicinal plants, *Azadirachta indica* A.Juss. was the most frequently used traditional medicinal plant against COVID-19-related symptoms followed by *Moringa oleifera* Lam., *Pyrenacantha kaurabassana* Baill., and *Sclerocarya birrea* (A. Rich.) Hochst. [37].

In Morocco, the most commonly used medicinal plant against COVID-19 was *Eucalyptus globulus* Labill., which was reported in 3 different studies [25, 32, 44]. *Eucalyptus globulus* Labill., its essential oils, and active compounds have antiviral effects and anti-infective properties [16, 30]. In addition, the Moroccan people living in different prefectures, are reported to use medicinal plants for the prevention and treatment of COVID-19. A total of 46 medicinal plants were identified, of which 25 species (most cited species: *Zingiber officinale* Roscoe, *Olea europaea* L., *Vitis vinifera* L., and *Allium sativum* L.) strengthen the immune system, 18 species (most cited species: *Eucalyptus globulus* Labill., *Trigonella foenum-graecum* L., and *Aloysia triphylla* (L'Hér.) Britton) relieve respiratory infection symptoms caused by SARS-CoV-2, and 11 species (most cited species: *Eucalyptus globulus* Labill., *Lavandula dentata* L., and *Eugenia caryophyllata* (L.) Alston) cleanse/sanitize the area [21]. Notably, polyherbal-based hydrotherapy obtained from African indigenous knowledge has been suggested to be used against COVID-19, including other infectious diseases in Nigeria [69].

As reported in Table 2, in Ethiopia, 32 herbal and non-herbal medicinal products and 15 plant species have been used in African traditional medicine to treat and prevent symptoms of COVID-19 [110]. In another study, a total of 111 plant species have been identified which are used to treat viral infections by the traditional healers of Ethiopia and are suggested as potential therapeutics against COVID-19 [103]. In West Africa, a total of 145 plant species have been identified that have been in use for the management of respiratory tract diseases during the COVID-19 pandemic, wherein *Guiera senegalensis* J.F.Gmel.*, Sterculia setigera* Delile*, Bridelia ferruginea* Benth.*, Euphorbia hirta* L., and *Terminalia avicennioides* Guill. & Perr. were the most cited plants [54].

In Cameroon, among 29 plant species, *Citrus aurantiifolia* (Christm.) Swingle*, Zingiber officinale* Roscoe*,* and *Allium sativum* L. were reported as the most commonly used for COVID-19 symptoms [108]. A recent review on Cameroonian traditional medicinal plants published on March 2021 has recorded a total of 230 plant species as the potential sources of COVID-19 therapies. Among these species, 90 have been reported for traditional uses against at least 3 common COVID-19 symptoms, 30 species have been confirmed for having anti-COVID-19 phytochemicals, and 10, 52, and 14 species for immunostimulatory, anti-inflammatory, and antiviral properties, respectively [48].

#### South American ethnomedicinal plants in COVID-19 healthcare

Peru has reported a total of 17 plant species that have been in use in the treatment of respiratory symptoms during the COVID-19 pandemic. Among these, the most commonly used plant species were *Eucalyptus globulus* Labill.*, Piper aduncum* L.*, Zingiber officinale* Roscoe*, Allium sativum* L.*, Matricaria recutita* L. [115].

#### Turkish ethnomedicinal plants in COVID-19 healthcare

In Turkey, a cross-sectional survey study was conducted among 389 participants wherein 153 (39.3%) participants reported to have used traditional and complementary medicine during COVID-19, especially, as herbal medicines and nutritional supplements such as vitamins [61]. Medicinal plants such as *Rosa canina* L., *Mentha* × *piperita* L., *Sambucus nigra* L., *Olea europaea* L., *Eucalyptus spp*., *Melissa officinalis* L., and *Origanum vulgare* L. have been used in Turkish folk medicine for centuries to treat flu and have been suggested for potential use in COVID-19 treatment [91].

#### Sub-Saharan Africa ethnomedicinal plants in COVID-19 healthcare

A total of 99 plant species have been used as traditional medicines against three common symptoms of COVID-19, such as fatigue, fever, and coughs in 16 sub-Saharan Africa countries. *Zingiber officinale* Roscoe, *Lippia javanica* (Burm.f.) Spreng., *Ocimum gratissimum* L., *Citrus limon* (L.) Osbeck, and *Artemisia afra* Jacq. ex Willd. were found to be the most commonly used medicinal plants in several countries of the region [116].

Overall, from the compilation of the data in Table 2, it has been observed that the most cited plant species (marked bold) that were used against COVID-19 symptoms in diverse geographical locations are a total of 16 plants species which were cited at least two times. All of these plants and their derivatives have possessed antiviral activity validated by in vitro*, *in vivo, and in silico pharmacological evaluations (see Table 3).

**Table 3:** Antiviral mechanisms of the most cited ethnomedicinal plants used for SARS-CoV-2 causing COVID-19 and associated symptoms reported from diverse geographical locations

Most cited ethnomedicinal plant species	Antiviral mechanisms (in vitro*, *in vivo, and in silico)
*Allium sativum* L.	Blocks viral entry into host cells, inhibits viral RNA polymerase, reverse transcriptase, DNA synthesis and immediate-early gene 1(IEG1) transcription, downregulates the extracellular-signal-regulated kinase (ERK)/mitogen-activated protein kinase (MAPK) signaling pathway [88]
*Andrographis paniculata* (Burm. f.) Nees	Fusion and adsorption of virus to the host cell, binding to viral receptor and coreceptor, enzymes involved in the DNA/RNA/Genome replication by the virus, translation, post-translation and reverse transcription [58],enhances cytotoxic T cells, natural killer (NK) cells, phagocytosis, and antibody-dependent cell-mediated cytotoxicity (ADCC) [51]
*Artemisia herba-alba* Asso	Phytochemicals (4,5-di-O-Caffeoylquinic acid, rutin, and schaftoside) of the plant has binding affinity to SARS-CoV-2 M^pro^ protein complex [55]
*Artemisia vulgaris* L.	Induction of cellular ROS, blunting the PI3K/Akt/p70S6K signaling pathway, binding to NF-kB/Sp1 or inducing an endocytosis inhibition mechanism, inhibition of TGF-β signaling [67]
*Azadirachta indica* A. Juss.	Blocks virus entry, inhibits viral binding to the cell [106], interacts with SARS-CoV-2 membrane (M) and envelope (E) protein [28]
*Camellia sinensis* (L.) Kuntze	Inhibits viral RNA replication, erythrocyte agglutination; blocks viral entry into hose cells; downregulates RNA synthesis; interferes core promoter transcription, virus absorption; prevents cell to cell transmission; disrupts viral membrane integrity; and reduces Nf-kB expression [119]
*Citrus limon* (L.) Osbeck	Hepatitis A virus titer was reduced by 2.84 log TCID50/ml after treatment with *citrus lemon* EO at 0.5% [20]
*Curcuma longa* L.	Inhibits SARS-CoV 3CL protease, hemagglutinin, viral RNA replication, viral assembly, virus binding to host cell; disrupts viral membrane proteins [104]
*Eucalyptus globulus* Labill.	Inhibits viral replication [30]
*Euphorbia hirta* L.	The extracts of the plant inhibit viral replication with IC50 values of 9 μg/ml (T1) and 5 μg/ml (T3) evaluated against HIV-1 [52]
*Glycyrrhiza glabra* L.	Blocks the SARS-CoV-2 replication (at a concentration of 0.5 mg/mL)) by inhibiting the viral main protease M^pro^ [113]
*Mangifera indica* L.	Inhibits viral replication and viral protease [12, 117]
*Nigella sativa* L.	Inhibits SARS-CoV-2 protease and ACE2 receptors, affinity with SARS-CoV-2 enzymes and proteins [129]
*Psidium guajava* L.	Inhibits early stage of viral infections and viral hemagglutination [102]
*Syzygium aromaticum* (L.) Merr. & L.M.Perry	Inhibits the viral DNA polymerase [38], immunomodulator [41]
*Zingiber officinale* Roscoe	Blocks viral attachment and internalization [34],inhibits viral replication [1]

### Traditional aromatherapy and essential oils for COVID-19 healthcare

Essential oils have been used for their pharmacological activities in different traditional medicines worldwide such as Arabian, Ayurvedic, Chinese, Iranian, Korean, American, and European Pharmacopoeias, and essential oils of *Eucalyptus, Mentha,* and *Citrus* have been registered for clinical trials [94]. Plant-derived essential oils or volatile oils have antiviral compounds which may aid in the management of COVID-19 symptoms [59, 122]. Essential oils of aromatic plants that have virucidal activity can be a hope for effective anti-COVID-19 therapy development because essential oils have been shown to disrupt the viral membrane proteins. In recent times, the antiviral and virucidal properties of essential oils and their compounds have been reviewed (refer to [85, 120]). Furthermore, Saleem and Ezzat [90] have discussed and given perspectives on the antiviral activity of some essential oils on COVID-19 aspects. Aromatherapy and compounds in essential oils can also reduce inflammation, irritation, muscle aches, ameliorate headaches, insomnia, and indigestion, and protect the respiratory system, as well [17].

Carvacrol, a bioactive compound in essential oils that belongs to many aromatic plants, exerts immunomodulatory, anti-inflammatory, and antiviral properties as well as can be potential for COVID-19 therapeutics development [60]. Compounds like anethole, cinnamaldehyde, carvacrol, geraniol, cinnamyl acetate, L-4-terpineol, thymol, and pulegone from essential oils of aromatic plants have the potential to inhibit spike proteins of the virus, assessed by molecular docking and the conceptual density functional theory [68]. Eucalyptol-rich essential oils can be useful remedies for patients with mild infections caused by COVID-19, notably against respiratory infections [111]. The possible mechanisms of essential oils from cinnamon to decrease oxidative stress and inflammation in patients with COVID-19 have been reviewed [132].

In silico approaches have also been employed to evaluate the potential antiviral efficacy of essential oil compounds against SARS-CoV-2 such as citronellol, alpha-terpineol, eucalyptol, d-limonene, 3-carene, o-cymene, and alpha-pinene found in essential oils of *Eucalyptus* and *Corymbia* species [78], as well as cuminal, carvacrol, myrtanol, and pinocarveol [126]. Essential oil compounds of Brazilian plant species that are used in traditional medicine have shown anti-COVID-19 potential by a molecular docking study, see Table 4 [13]. In addition, two controlled clinical trials have been registered to assess the efficacy of plant-derived essential oils against asymptomatic and symptomatic COVID-19 patients (*Trial ID: CTRI/2021/07/034962*) in India, and post-COVID-19 fatigue (*ClinicalTrials.gov identifier: NCT04980573*) in the United States. Above all else, active constituents in essential oils such as phenols, terpenoids, alkaloids, and phenylpropanoids possess potent antiviral properties and are safe for human use with fewer side effects [62].

**Table 4:** A list of identified essential oil compounds showed anti-COVID-19 efficacy by a molecular docking study (compiled from [13]

Identified essential oil compound	Plant source	Part (s) used
(*E*)-α-atlantone	*Cordia curassavica* (Jacq.) Roem. & Schult.*, Lippia alba* (Mill.) N.E.Br. ex Britton & P.Wilson*, Schinus terebinthifolia* Raddi	Leaf
Allo-aromadendrene epoxide	*Lippia alba* (Mill.) N.E.Br. ex Britton & P.Wilson	Leaf
Amorpha-4,9-dien-2-ol	*Aloysia gratissima* (Gillies & Hook.) Tronc.	Leaf and branch
Azulenol	*Lantana camara* L	Leaf
Germacrene A	*Aloysia gratissima* (Gillies & Hook.) Tronc.*, Croton heliotropiifolius* Kunth*, Lantana camara* L.*, Lippia alba* (Mill.) N.E.Br. ex Britton & P.Wilson*, Lippia origanoides* Kunth*, Schinus terebinthifolia* Raddi	Leaf, aerial part
Guaia-6,9-diene	*Lantana camara* L.	Aerial part
Hedycaryol	*Lippia alba* (Mill.) N.E.Br. ex Britton & P.Wilson	Aerial part
	*Schinus terebinthifolia* Raddi	Ripe fruit a seed
Humulene epoxide II	*Aloysia gratissima* (Gillies & Hook.) Tronc.	Leaf and branch
	*Lippia origanoides* Kunth	Leaf and florescence
α-Amorphene	*Lippia alba* (Mill.) N.E.Br. ex Britton & P.Wilson	Leaf, stalk, flower
α-Cadinene	*Lippia alba* (Mill.) N.E.Br. ex Britton & P.Wilson	Leaf, stalk, flower
	*Lippia origanoides* Kunth	Flower, leaf, stem, and inflorescence
α-Calacorene	*Lippia origanoides* Kunth	Flower, leaf, stem
α-Muurolene	*Cordia curassavica* (Jacq.) Roem. & Schult.	Leaf
	*Lippia alba* (Mill.) N.E.Br. ex Britton & P.Wilson	Leaf, fin branch
	*Lippia origanoides* Kunth	Leaf and florescence
	*Lantana camara* L.	Aerial part
Aristochene	Not specified	–

### Advances in traditional phytomedicines and their purported prophylactic and pharmacological effects on SARS-CoV-2

#### Role of ethnomedicinal plants for in silico evaluation against SARS-CoV-2

The antiviral activity of the extracts of medicinal plants and their bioactive compounds have been confirmed by in vitro*, *in vivo*,* and in silico assays to inhibit direct entry of the SARS-CoV-2 and ACE2 receptors as well as block the serine protease TMPRRS2. It is also reported that medicinal plants can inhibit papain-like or chymotrypsin-like proteases that are required in the life cycle of SARS-CoV-2 [4, 24]. Several plant biometabolites including extracts have been reported previously for in vivo and in vitro anti-coronavirus properties such as lectins, saikosaponins, emodin, quercetin derivatives, lectin, ethanolic, and water extract of the plant parts or whole plant [81]. Traditional medicinal plants and their secondary metabolites have been evaluated by an in silico approach against coronavirus for the inhibition of hACE2 receptor that facilitates virus entry into host cells, also studied for inhibition of non-structural proteins 3CL^pro^ and PL^pro^ [33].

Molecular docking was conducted to investigate compounds of selected South African traditional medicinal plants and their potential inhibitory activities against SARS-CoV-2 RDRP, 3CL^pro^, and SARS-CoV-2 RBD. Among those investigated were arabic acid, which was isolated from *Acacia senegal* (L.) Willd.; and L-canavanine from *Sutherlandia frutescens* (L.) R.Br., reported for inhibition of SARS-CoV-2 3C-like main protease; and hypoxoside from *Hypoxis hemerocallidea* Fisch., C.A.Mey. & Avé-Lall. and uzarin from *Xysmalobium undulatum* (L.) W.T.Aiton, which were reported for inhibition of SARS-CoV-2 RBD and SARS-CoV-2 RNA-dependent polymerase [43]. In addition, Attah and colleagues reviewed evidence-based antiviral and anti-SARS-CoV-2 properties of African traditional medicinal plants on the basis of in silico*, *in vitro, and in vivo approaches [18].

Phytochemicals of 3 Indian traditional medicinal plants, *Mentha arvensis* L., *Coriandrum sativum* L., and *Ocimum sanctum* L., were found to exhibit in silico high binding affinity to the nucleocapsid phosphoprotein (N) of SARS-CoV-2, which has a key role in replication, transcription, and viral genome assembly [74]. A molecular docking analysis conducted by Khan and coauthors to evaluate anti-SARS-CoV-2 activity showed that EGCG (epigallocatechin gallate) exhibits very strong interactions through binding energies of − 9.30, − 8.66, and − 8.38, kcal/mole with the free enzyme of main protease ( 6Y2E), chimeric receptor-binding domain complexed with human ACE2 ( 6VW1), and NSP15 endoribonuclease ( 6VWW) encoded proteins of SARS-CoV-2, respectively [66]. In a literature review study, an *Angelica keiskei* (Miq.) Koidz-derived bioactive compound, xanthoangelol E, showed strong inhibitory activity with IC50 value of 1.2 μM against SARS-CoV PL^pro^, and also quercetin was indicated as being a promising antiviral bioactive compound [76]. Bioactive compounds of medicinal plants of Indian origin such as Cucurbitacin E and orientin can be promising candidates for the development of COVID-19 therapeutics, investigated against the structural targets of SARS-CoV-2 (M^pro^ and spike protein) and human ACE2 receptor [7].

**Fig. 1:**
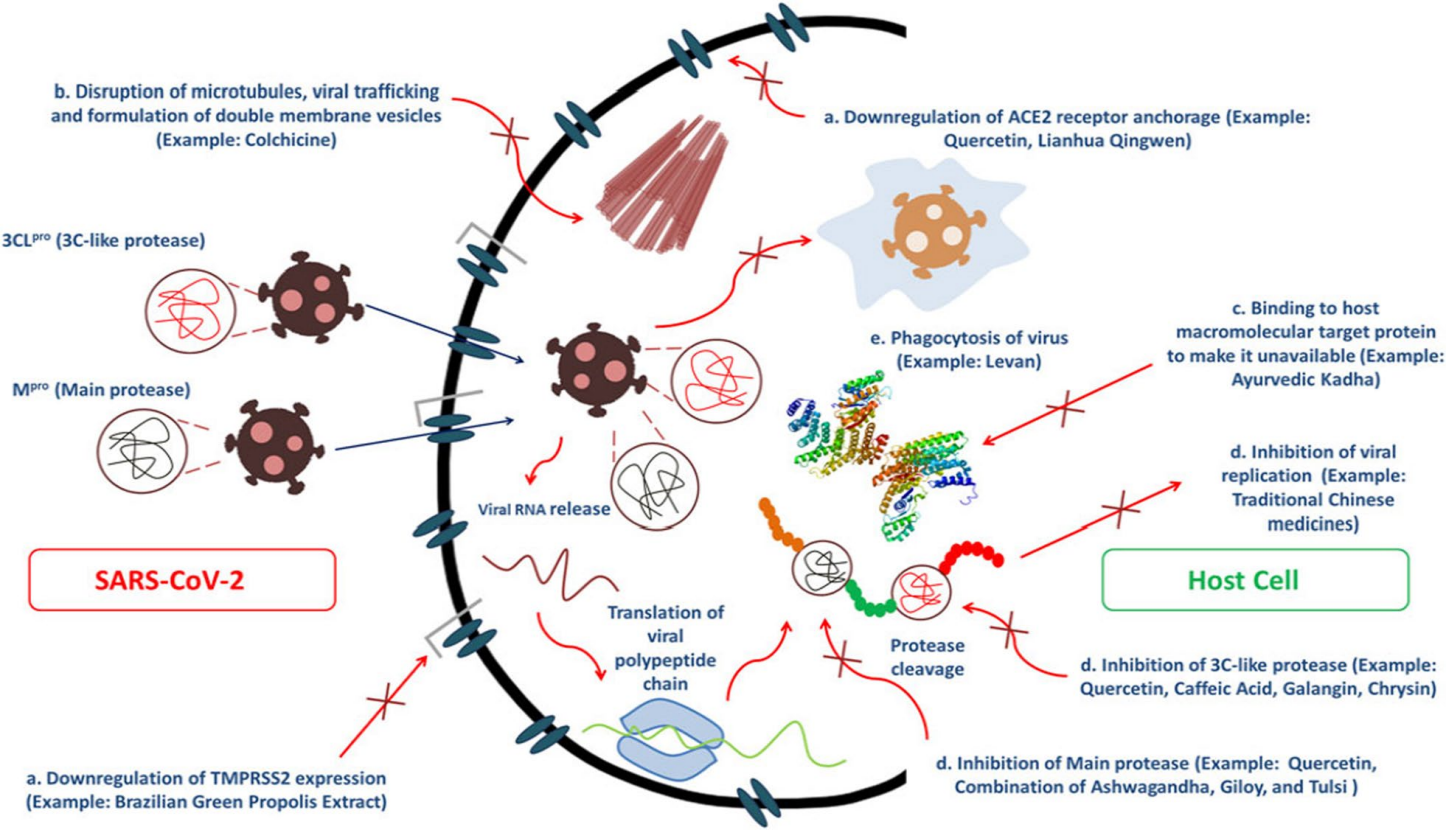
Several mechanisms of actions of traditional phytomedicines and phytoconstituents to perform anti-SARS-CoV-2 efficacy. “(A) inhibition of main protease and 3C-like protease, (B) Disruption of microtubules, viral trafficking and formulation of double membrane vesicles, (C) Binding affinity toward host macromolecular target protein to make it unavailable and (D) Downregulation of ACE2 receptor anchorage and TMPRSS2 expression which ultimately causes inhibition of viral replication.” (Figure with legend adapted from Alam et al. [9])

Phytoconstituents of *Withania somnifera* (L.) Dunal, *Tinospora cordifolia* (Willd.) Miers, and *Asparagus racemosus* Willd. that have been proposed to play role in the traditional medicine application of Ayurveda, as a “Rasayana” for rejuvenation by boosting the immune system, have been assessed in silico for immunomodulatory potential as therapeutic adjuvants in COVID-19 management [29]. Rivero-Segura and Gomez-Verjan [87] have performed an in silico screening of 100 phytochemicals isolated from the most commonly used medicinal plants of Mexico for the SARS-CoV-2, and found ten compounds (emodin anthrone, kaempferol, quercetin, aesculin, cichoriin, luteolin, matricin, riolozatrione, monocaffeoyl tartaric acid, aucubin) that meet lead-likeness criteria. Figure 1 demonstrates several mechanisms of actions of traditional phytomedicines and phytoconstituents to perform anti-SARS-CoV-2 efficacy.

#### Role of ethnomedicinal plants in clinical trials for the treatment of COVID-19

It is worth mentioning that the efficacy of 32 Indian traditional medicinal plants against COVID-19 is registered for controlled clinical trial investigation which includes species, such as *Tinospora cordifolia* (Willd.) Miers stem, *Piper longum* L. fruit, ZingiVir (polyherbo-mineral drug), Chyawanprash (an Ayurvedic health supplement which is made up of a super-concentrated blend of nutrient-rich herbs and minerals), Aayudh Advance (an herbal formulation consisting of essential oils), and Ashwagandha (*W. somnifera*) (see [98]). Another clinical trial has also been registered (trial no: PACTR202012898601759) to evaluate the antiviral effect of the seed extract of *Silybum marianum* (L.) Gaertn. for the treatment of patients with COVID-19 in Nigeria (https://pactr.samrc.ac.za/TrialDisplay.aspx?TrialID=14547). Milk thistle or *Silybum marianum* (L.) Gaertn. is a common herbal medicine species used around the world with wide ethnomedicinal usage and good clinical support [131].

A phytomedicine named “Cospherunate,” which is a combination of 50 mg Artesunate/125 mg Amodiaquine (*Artemisia spp.* and *Artemisia* derivatives), has been registered for clinical trials (*ClinicalTrials.gov Identifier: NCT04502342*) in therapy for COVID-19 treatment in Guinea. Additionally, other registered clinical trial interventions using *Artemisia spp.* and *Artemisia* derivatives in COVID-19 therapy have been listed [77]. *Artemisia vulgaris* L., that is a traditional Chinese herb, has been reported to exhibit numerous potential health benefits [100].

In Thailand**,** *Andrographis paniculata* (Burm. f.) Nees has been approved for a pilot clinical trial in the treatment of mild COVID-19 symptoms [26], and another traditional herb, *Boesenbergia rotunda* (L.) Mansf., known as finger root, has been planned for human trial as the active ingredients in the plant were reported to eliminate SAS-CoV-2 in hamster experiments [123].

In Iran, the Food and Drug Administration has approved the use of four traditional herbal medicines in the treatment of COVID-19 [46], and clinical studies of licorice extracts, mallow (*Malva sylvestris* L.), echinacea, ginger, mixed with sage, fennel, and St. John’s wort, and Fluherb (grass extracts, plantain, isop, licorice, and turmeric, suspension) have been carried out to investigate effects in COVID-19 therapy [50]. Furthermore, the herbal medicine Xagrotin, which is a combination of several local plants from the Zagros Mountains located in Kurdistan, has entered into clinical trial to evaluate outcome of patients with early-stage COVID-19 (https://clinicaltrials.gov/ct2/show/NCT05017493).

#### Role of ethnomedicinal plants in human gut microbiome against COVID-19

Targeting gut microbiota (GM) to prevent and treat COVID-19 has been viewed as an alternative strategy [101] as GM plays an important role in the maintenance of host homeostasis, and GM can modulate ACE2 which is expressed in intestinal cells and is crucial for the entry of SARS-CoV-2 into the host cells [133]. Dysbiosis of GM or altered oral microbiome has a correlation with the severity and hospitalization of COVID-19 in patients compared to healthy individuals [125, 130] as well to produce pro-inflammatory cytokines [5]. A systematic review has found imbalanced intestinal and respiratory microbiomes in hospitalized COVID-19 patients [127]. Hence, COVID-19, as well as the post-COVID-19 gastrointestinal (GI) complications, has bidirectional interactions between the gut and lung microbial dysbiosis [36].

**Fig. 2:**
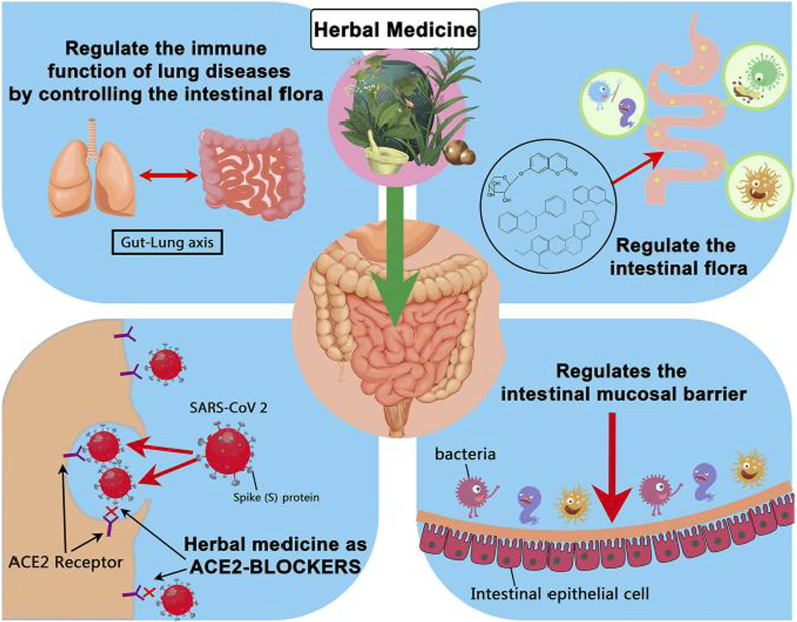
Summary of possible ways in which herbal medicines can affect the prognosis of COVID-19 by regulating the intestinal flora (adapted from Chen et al. [35])

Medicinal plants that have supportive roles in immune stimulation and maintenance of a balanced gut microbiome may be effective strategies for the management of COVID-19 [35] as a healthy gut microbiota is essential for a strong antiviral immunity [8]. However, plant-based ethnomedicines are mostly ingested orally, and the efficacy of medicinal plants is regulated by the role of GM or GM-derived metabolites [105, 118]. Figure 2 illustrates how medicinal plants and the gut microbiome have potential roles in the attenuation of SARS-CoV-2 causing COVID-19. In general, orally administrated ethnomedicinal plants or ethnomedicinal phytochemicals are digested by gut microbiota-derived digestive enzymes (β-glucuronidase, β-glucosidase, and galactosidase) that facilitate biotransformation into the absorbable bioactive components with potent bioactivities and then enter the body and induce pharmacological effects [14, 124]. Hence, traditional phytomedicines have the ability to change the composition of GM and its secretions which later induce physiochemical changes as well [14].

It is worth noting that the potential role of GM in traditional Chinese herbal medicines for the attenuation of SARS-CoV-2 has been demonstrated earlier (see [133]). On the other hand, Chen et al. have reviewed and listed the roles of medicinal plants to include ACE2 inhibition in the GI tract, support in restoration of the GI flora for the regulation of intestinal mucosal barrier and respiratory immunity, and the attenuators of the specific complexities that caused by COVID-19 disease [35].

#### Role of ethnomedicinal plants as immunomodulation against COVID-19

**Fig. 3:**
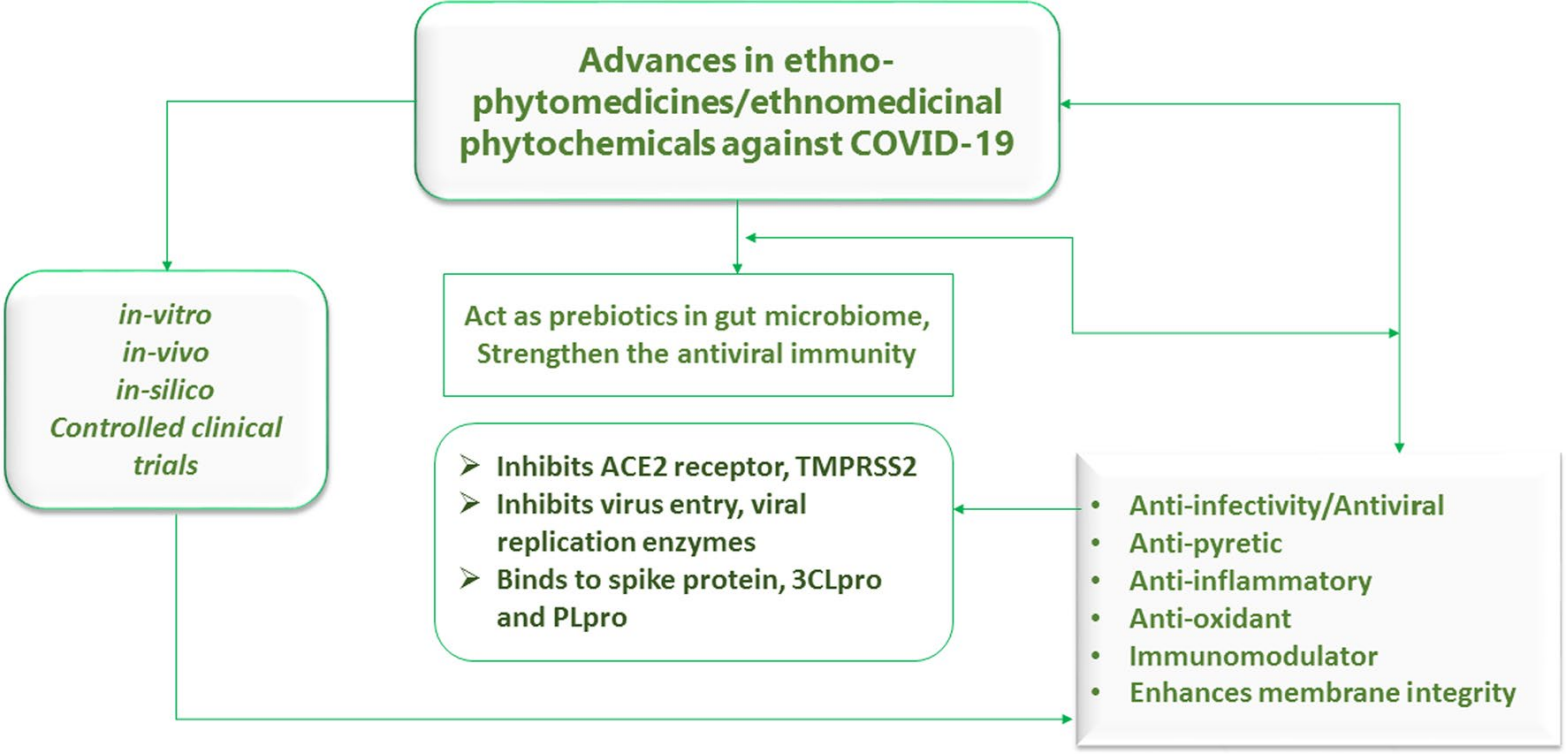
Summary of advances in ethno-phytomedicinal knowledge in the terms of COVID-19 healthcare management

As ethnomedicinal plants are potential and promising sources of immunomodulators against COVID-19, ethnomedicinal plants such as *Panax ginseng* C.A.Mey., *Achillea millefolium* L., *Andrographis paniculata* (Burm. f.) Nees, *Asperagus racemosus* Willd., *Bauhinia variegate* L*., Hyptis suaveolens* (L.) Poit., *Cleome gynandra* L., *Alternanthera tenella* Colla, *Abutilon indicum* (L.) Sweet, *Heracleum persicum* Desf. ex Fisch., C.A.Mey. & Avé-Lall., *Lagenaria siceraria* (Molina) Standl., and *Chlorophytum borivilianum* Santapau & R.R.Fern. are considered immunomodulators and reported for enhancing the body’s stamina to fight against COVID-19 [107]. Notably, Silveira and colleagues have identified a total of 39 herbal medicines that were found to be very promising for COVID-19 patients, and out of 39 species they suggested *Althaea officinalis* L., *Commiphora molmol* (Engl.) Engl. ex Tschirch, *Glycyrrhiza glabra* L., *Hedera helix* L., and *Sambucus nigra* L. for safety and use as adjuvants in the treatment of early/mild symptoms of COVID-19 [96]. The mechanisms underlying immunomodulatory activities of medicinal plants and their bioactive phytoconstituents are (1): activation of NK cells, macrophages, neutrophils and eosinophils, (2): simulation of signaling molecules (NF-kB, JNK, P38, ERK), (3): expression of cytokines (TNF-α, IL-6, IL-8), (4): expression of chemokines (CCL4, CCL8), (5): promotion of T-lymphocytes, transformation to T-helper cells, (6): inhibition of pro-inflammatory chemicals [10]. Figure 3 portrays the recent advances in traditional phytomedicines against SARS-CoV-2.

## Conclusions

Ang et al. [15] have found 9 guidelines of herbal formulas (mostly based on clinical results) recommended for the medical observation period of COVID-19, of which 8 were issued by the Chinese provincial government for 12 herbal formulas and 1 was issued by the Korean Pulmonary Association of Traditional Medicine for 2 herbal formulas. Antiviral traditional medicinal plants have broad-spectrum activities such as viral attachment inhibition of the host cell, viral replication inhibition, downregulation of translation of viral proteins and virus-induced pro-inflammatory/inflammatory signaling pathways in the host cell, suppression of RNA replication, as well as activities as antioxidants to inhibit virus-induced oxidative stress [45].

Numerous in silico studies on traditional medicinal plants and secondary metabolites have been performed in the search for potent COVID-19 therapeutics, of which only a few have in vitro or in vivo antiviral activity; some of these are registered for controlled clinical trials. The reliance on ethnomedicinal plants is suddenly advanced due to the outbreak of a new coronavirus, SARS-CoV-2, as medicinal plants can target and inhibit viral 3CL^Pro^, spike proteins, main protease, RDRP, and host TMPRSS2 protease, ACE2 receptor. Moreover, ethno-phytomedicine has roles in the gut microbiome and immunomodulatory activity for the potential biotherapeutic efficacy against COVID-19. We have identified 16 plant species (*A. sativum, A. paniculata, A. herba-alba, A. vulgaris, A. indica, C. sinensis, C. limon, C. longa, E. globulus, E. hirta, G. glabra, M. indica, N. sativa, P. guajava, S. aromaticum,* and *Z. officinale*) that are the most commonly used traditional phytomedicine for COVID-19 prevention and treatment in various geographical locations. These plant species have reported in vitro, in vivo, and in silico antiviral efficacy with diverse mechanisms (such as inhibition or blocking viral entry into host cells, viral RNA polymerase and RNA replication, viral DNA synthesis, viral hemagglutination, viral assembly, disruption of viral membrane proteins, enhancing cytotoxic T cells, natural killer (NK) cells, and phagocytosis). Due to these advances in understanding, there should be enhanced recognition for extensive biological research of ethnomedicines for developing plant-based COVID-19 eradication strategies.

According to the above discussions, the necessity to build collaborations among those with ethnomedicinal plants knowledge, scientists, and technologists to develop the most efficient and the safest adjuvant therapeutics against the pandemic of the twenty-first century, COVID-19, has been realized.

## Data Availability

Not applicable.

## Abbreviations

ACE2: Angiotensin-converting enzyme 2
TMPRSS2: Transmembrane serine protease 2
3CL^Pro^/M^pro^: 3-Chymotrypsin-like protease
PL^Pro^: Papain-like protease
RBD: Receptor-binding domain
RDRP: RNA-dependent RNA polymerase
ADMET: Absorption, distribution, metabolism, excretion, and toxicity
GM: Gut microbiota
GI: Gastrointestinal
